# Examining the relationship between non-suicidal self-injury and mental health among female Arab minority students: the role of identity conflict and acculturation stress

**DOI:** 10.3389/fpsyt.2023.1247175

**Published:** 2023-11-07

**Authors:** Sahar Meisler, Sheren Sleman, Michal Orgler, Inbal Tossman, Sami Hamdan

**Affiliations:** The School of Behavioral Sciences, The Academic College of Tel Aviv-Yafo, Tel Aviv-Yafo, Israel

**Keywords:** non-suicidal self-injury, identity conflict, depression, risky substance use behaviors, acculturation stress

## Abstract

**Background and objective:**

Research suggests that individuals from minority backgrounds, including immigrants and ethnic minorities, may be at a higher risk for non-suicidal self-injury (NSSI). The aim of the present research is to examine the relationship between non-suicidal self-injury (NSSI) and identity conflict and acculturation stress, depression, and risk behaviors among female Arab minority students.

**Methods:**

The sample analyzed consisted of 1,529 female Arab students (85.8% B.A. students, 14% M.A. students) aged 21–54 (*M* = 23, SD = 4.17). The participants completed self-reported questionnaires assessing NSSI engagement, identity conflict and acculturation stress, depression, and risky substance use behavior.

**Results:**

As expected, we found a significant positive association between NSSI and identity crisis. In addition, an identity conflict and acculturation stress impact the effect of depression and risky substance use behaviors in engaging in self-injury. Namely, participants were more likely to engage in NSSI when they also experienced identity conflict and acculturation stress and exhibited depressive symptoms and tended to display risky substance use behavior.

**Conclusion:**

These findings provide evidence that the relationship between depression, risky substance use behavior, and NSSI may be stronger among individuals who experience higher levels of identity conflict and acculturation stress. Implications for intervention and future research are briefly presented.

## 1. Introduction

Non-suicidal self-injury (NSSI) behavior is a growing worldwide clinical and public health concern. NSSI is defined as the direct and deliberate destruction of one’s own bodily tissue (for example, by cutting, burning, or scratching oneself badly) in the absence of the intent to die and for reasons not socially sanctioned (1, 2). NSSI is affected by cultural and economic conditions and life experiences that may lead to such behavior. The literature has consistently demonstrated that the young adult population is at increased risk for self-injury compared to the general population (3, 4). Researchers and clinicians emphasize the increased magnitude of NSSI as a psychological and physiological health risk for young adults, thus increasingly devoting research attention to this phenomenon (5). For instance, a meta-analysis estimated that 13% of young adults (aged 18–24) have engaged in self-injury at some point in their lives (6).

Self-injury may be linked to numerous psychiatric disorders (5, 7) including major depressive disorder (MDD), which is a significant predictor of future NSSI (8, 9). For example, in Australia, among 12–17-year-olds with depression, 47% reported non-suicidal self-harm over the course of 1 year. In contrast, among 12–17 years nonclinical adolescents, only 4.2% report engaging in non-suicidal self-harm behavior (10). Some studies suggest that people may choose to engage in NSSI to reduce feelings of numbness or emptiness (11, 12), which are emotions that also characterize a depressive phase (13). In other words, the action of self-harm is expected to lead to relief from the negative emotions that accompany depression.

NSSI has also been associated with risky substance use behavior, such as alcohol and cannabis use, especially among young adults (14). Like NSSI, which is used to reduce aversive stress (15), alcohol has been found to reduce various negative emotions (16). Additionally, cannabis use was associated with an increased prevalence of self-injury (17). Mechanisms underlying this relationship suggest the role of shared genes and a family environment (18).

People who identify as members of a minority group are typically more likely to engage in NSSI (19). Minority stress, as defined by minority stress theory (20), has been directly associated with increased NSSI risk in ethnic minorities due to an identity conflict (21). Identity conflict is perceived as an incompatibility between two or more of an individual’s identity domains (22). Even though holding multiple identities could increase well-being and predict successful adjustment following life changes (23), there may be a dissonance between the meanings of one’s different identities that may be threatening and challenging to deal with (24). Accordingly, heightened levels of anxiety and depression were found among bicultural people who experienced conflict between their distinct cultural identities (25).

Social structure inequities can lead to increased stress caused by acts of discrimination and social exclusion, which are added burdens that socially advantaged groups are not equally exposed to (26). The assumption is that the decreased social standing of stigmatized minority groups could lead to individuals’ increased exposure to stressful life situations and social inequality. In addition, such groups have fewer resources to cope with such events. The cognitive-emotional model of NSSI (27) suggests that emotional reactivity affects how individuals interact with the world. In ethnic minorities, such emotional reactivity may result from earlier life experiences, such as stressful social environments. These experiences may be avoided or modulated through the use of NSSI.

In the current study, the population being studied is the Female Arab minority student’s population in Israel. As a result of the process of westernization within Israel, the Arab minority in Israel is often referred to as a society in transition, caught between Eastern and Western cultures. They speak a different language (Arabic) compared to the majority group’s language (Hebrew), have other religions (rather than Judaism)—most Arabs in Israel are Muslims—and preserve an autonomous cultural existence (28). Moreover, as minorities in the context of the Israeli-Palestinian conflict, Palestinian citizens of Israel have not been treated as equal citizens in many respects, such as accessing medical services and workforce participation (29). This reality has significant implications for the mental health of this population. For example, Arab citizens of every demographic, socioeconomic status, and state of health are more likely to report a lower level of life satisfaction along with a higher level of feelings of loneliness than Jews (30).

The results of NSSI by gender are mixed. Yet, evidence has been found attesting to a higher prevalence of NSSI among females compared to males (31–33). Some hypotheses for why women are at greater risk of self-harm than men are, for example, due to their greater propensity for depression (34). Another possibility is gender socialization of emotions that may impact the type of emotions men and women experience in a way that leads women to be more likely to engage in NSSI [e.g., shame vs. anger; (35)]. Thus, because women are at higher risk of engaging in NSSI than men, we chose to focus on young women in this study.

To date, several studies have examined the association between identity conflict, acculturation stress and NSSI among ethnic minorities (36–38). However, these studies did not examine these associations in the presence of depressive symptoms and risky substance use behaviors. In the context of our study, it was vital to focus on a population that presents a unique intersection of gender and ethnic identity—the female Arab minority students in Israel. Several factors informed this choice: (1) There is a noticeable gap in studies that explore NSSI in this population. (2) as discussed, the Arab minority in Israel undergoes distinctive cultural changes, often referred to as a society in transition, toggling between Eastern and Western influences. (3) For Arab women, attending university in Israel signifies an educational journey and a cultural shift laden with potential identity conflicts and stressors. Considering these considerations, our study was designed to provide an essential understanding of NSSI among female Arab minority students in Israel.

Additionally, the research on identity conflict, acculturation stress, and NSSI has mainly been investigated among gender and ethnic minorities in the United States. Therefore, this study aims to examine the association between NSSI, identity conflict and, acculturation stress, depression, and risky substance use behavior as presented by alcohol misuse and cannabis use. We hypothesized that (1) there would be a significant association between identity conflict and acculturation stress and engaging in NSSI, depression, and risky substance use behaviors; (2) a significant positive association would be observed between depression and NSSI; (3) a significant positive association would be found between risky substance use behavior and NSSI; and (4) Identity conflict and acculturation stress would impact the effect of depression and risky substance use behavior on NSSI.

## 2. Method

### 2.1. Participants

The study included 1,529 female Arab students (85.8% B.A. students, 14% M.A. students) aged 21–54 (*M* = 23, SD = 4.17). Participants defined themselves as either religious (12.5%), traditional (69%), or secular (17.4%), and they studied in several academic institutions in Israel.

### 2.2. Procedure

The participants were recruited by (1) advertising on social media, (2) face-to-face recruitment in colleges and universities, and (3) using the snowball method. All participants were informed about the aim of the study and were directed to an online webpage and were asked to complete the questionnaires anonymously. They were also provided with links to local mental health resources. The inclusion criteria for our study were: (A) Self-identified female Arab students (B) Currently enrolled in academic institutions in Israel and (C) Aged between 21 and 54 years. Exclusion criteria included: (A) Individuals not currently enrolled in an academic institution and (B) Those outside the age bracket we specified. Participation was voluntary and were not monetarily compensated for participating in this study. The study was approved by the Institutional Review Board (IRB) at the Academic College of Tel-Aviv-Jaffa.

### 2.3. Measures

#### 2.3.1. Patient health questionnaire-9

A nine-item self-report questionnaire aimed to assess the severity of depression (39). The questionnaire assesses how often the subjects had been disturbed by any of the nine items during the immediately preceding 2 weeks (e.g., “Little interest or pleasure in doing things”). Each item is rated on a 4-point Likert scale (*0 = not at all; 1 = several days; 2 = more than half the days; and 3 = nearly every day*). The total score ranges from 0 to 27, with higher scores indicating greater severity of depression. In our study, we used a cut-off point of 10. Thus, those who received a score of 10 or higher were considered to be suffering from depression. We used the Arabic version of PHQ-9, and the internal consistency in this study was α = 0.90.

#### 2.3.2. The deliberate self-harm inventory

The DSHI is a self-report measure that assesses the lifetime history of various aspects of DSH [DSHI-Y; (40)]. The DSHI assesses participants’ intentionality (i.e., whether the self-harm was inflicted on purposes) and specifies the damage affected to tissue (e.g., “Have you ever intentionally severely scratched yourself, to the extent that scarring or bleeding occurred? If yes, how many times have you done this?”). Each item on the measure is rated on a 5-point Likert-type scale (*1 = No; 2 = Yes; 3 = Yes, 2–5 times; 4 = Yes, 6–10 times; 5 = Yes, more than 10 times*). This scale is used as a dichotomous variable to distinguish self-harming participants who frequently engaged in DSH from self-harming participants who engaged in DSH infrequently. Participants who reported engaging in NSSI at least one time were assigned a score of “1,” whereas those who did not report engaging in NSSI were assigned a score of “0”. The internal consistency in this study was α = 0.73.

#### 2.3.3. The CRAFFT (car, relax, alone, forget, friends, trouble) questionnaire − 2.0 version

This 4-item clinical assessment tool is designed to screen for substance-related risks (alcohol or cannabis use) and subsequent problems (e.g., “*During the past 12 Months, on how many days did you drink more than a few sips of beer, wine, or any drink containing alcohol*?”) (41). The answer options are dichotomous (Yes/No); Each “*Yes*” answer is scored as “1” and a total score of two or higher identifies “high risk” for substance use disorder. The internal consistency in this study was α = 0.74.

#### 2.3.4. Acculturation stress scale – revised

The Social, Attitudinal, Familial and Environmental Acculturation Stress Scale [SAFE-R; (42)] is a self-report questionnaire aimed to assess negative stressors experienced by both immigrant and later-generation individuals as they acculturate to the host culture (e.g., “I do not feel at home”). Participants are asked to rate the extent to which they perceive 24 items to be stressful in their lives on a 5-point Likert-type scale ranging from 0 (“Have not experienced stress”) to 5 (“Extremely stressful”), with higher scores indicating higher levels of acculturative stress. In our study, we used a cut-off point of 53 and the internal consistency in this study was α = 0.89.

#### 2.3.5. Demographics variables

Demographic information about age, marital status, parents’ residence, religiosity, and psychological treatment history were also obtained.

### 2.4. Data analysis

Statistical analyses were conducted with IBM SPSS Statistics version 27.0. Participants with and without NSSI were compared regarding demographic and clinical characteristics using *t*-tests or chi-square as appropriate. Logistic regression was used to analyze variables that differed significantly between those with and without NSSI. The selection of these variables was based on their consistent associations with NSSI in the existing literature and their statistical significance. Specifically, *depression*, *identity conflict*, *acculturative stress, risky substance use behavior* and *religiosity* were chosen based on their consistent associations with NSSI in the existing literature and their statistical significance in the analyses. In order to maintain coherence and avoid multicollinearity, additional demographic variables that showed statistical associations with NSSI were omitted (including the variables “*psychotherapy*” and “*medication*”). Finally, confirmatory path analysis models were conducted to identify and describe pathways to NSSI. Models were compared, and the parsimony model was chosen based on goodness of fit statistics, which includes comparison fit index (CFI), incremental fit index (IFI), and root mean square error of approximation (RMSEA). The alpha value was set to 0.05.

## 3. Results

Table 1 represents the characteristics of the study population, which included 1,529 participants aged 22–54 years. The sample included participants from three religions and of different levels of religiosity: Muslim (*N* = 1,230, 80%), Christian (*N* = 187, 12.3%) and Druse (*N* = 90, 5.9%), 85.8% are B.A. students (*N* = 1,313) and 14% M.A. students (*N* = 215). Clinically, 33.6% of the sample reported NSSI (*N* = 513). Additionally, about half of the sample (*N* = 668, 43.7%) reported depression, and 11.2% reported risky substance use behavior (*N* = 168).

**Table 1:** Demographic and clinical characteristics of the sample (*N* = 1,529).

Age (*M*; SD)	23	4.17
	*N*	%
Gender (female)	1,529	100
**Ethnicity**		
Muslim	1,230	80
Christian	187	12.3
Druse	90	5.9
**Religiosity**		
Religious	192	12.5
Traditional	1,070	69
Secular	267	17.4
**Marital status**		
Married	209	13.6
Single	1,306	85
Divorced	13	0.08
**Parents’ settlement pattern**		
Arab village	830	50.4
Arab city	121	8
Mixed city	587	38.3
**Residence**		
Dorms	347	22.6
Rented apartment	364	23.8
Parents’ house	818	53.4
**Education**		
Bachelor’s degree	1,313	85.8
Master’s degree	215	14
**Year of study**		
First-year	567	37
Second year	419	27.4
Third year	306	20
Fourth year	237	15.5
**Psychotherapy**		
Yes	215	14.1
**Medication**		
Yes	72	4.7
**Clinical characteristics**		
NSSI	513	33.6
Depression	668	43.7
Identity conflict and acculturation stress	793	52
Risky substance use behavior	168	11.2

The term mixed city refers to cities in which the population includes both a significant number of Jewish and Arab residents (43).

Table 2 displays the extent of engagement in Non-Suicidal Self-Injury (NSSI) by injury type among participants who have partaken in NSSI (*N* = 513). The most frequently observed type of injury is cutting (*N* = 250, 43.8%), whereas self-biting is the least prevalent (*N* = 10, 0.19%). Notably, for the majority of injury types, the predominant frequency of engagement is one time; however, in the case of self-biting, the most common frequency is more than 10 times (*N* = 6, 60%).

**Table 2:** Severity of NSSI engagement by type of injury among participants engaging NSSI (*N* = 513).

	1 time	2–5 times	6–10 times	<10 times	*N* (%)
Cutting	132 (52.8%)	87 (34.8%)	13 (5.2%)	18 (7.2%)	250 (43.8)
Burning	33 (86.8%)	5 (13.2%)	0	0	38 (19)
Scratching	81 (47.4%)	59 (34.5%)	18 (10.5%)	13 (7.6%)	171 (33)
Self-biting	0	0	4 (40%)	6 (60%)	10 (0.19)
Banging head/body	127 (54.3%)	78 (33.3%)	12 (5.1%)	17 (7.3%)	234 (45)
Self-punching	123 (57.5%)	72 (33.6%)	9 (4.2%)	10 (4.7%)	214 (41.4)

There are participants who engaged in various types.

Table 3 presents a comparison between the two study groups: those with NSSI and those with no engagement with NSSI. Participants who engaged in NSSI reported significantly higher depression (*t* = 11.473, *p* < 0.001), and identity conflict and acculturative stress (*t* = 8.496, *p* < 0.001) compared to those who did not report. Regarding risky substance use behavior, those with no NSSI behavior reported more risky substance use behaviors compared to those with no NSSI (χ^2^ = 33.451, *p* < 0.001). There were no differences in terms of marital status, parents’ settlement pattern, residence, and degree.

**Table 3:** Demographic and clinical characteristics by NSSI.

	Without NSSI history (*N* = 1,016)	NSSI history (*N* = 513)	Test	df	*p*
Age (*M* ± SD)	22.62 ± 3.87	23.14 ± 4.30	*t* = −2.318	1,523	0.021
**Ethnicity; % (N)**					
Muslim	79% (801)^a^	84% (429)^b^	χ^2^ = 15.264	3	0.002
Christian	14% (140)^a^	10% (47)^b^			
Druse	7% (66)^a^	4.6% (24)^a^			
**Religiosity; % (N)**					
Religious	14% (141)^a^	10% (51)^b^	χ^2^ = 12.989	2	0.002
Traditional	70% (720)^a^	68.2% (350)^a^			
Secular	15.2% (155)^a^	22% (112)^a^			
**Marital status; % (N)**					
Married	13.7% (139)	13.6% (70)	χ^2^ = 0.44	2	0.978
Single	85.4% (868)	85% (438)			
Divorced	0.9% (9)	0.7% (4)			
**Residence**					
Dorms	22% (225)	23.7% (122)	χ^2^ = 2.517	2	0.284
Rented apartment	23% (233)	25.5% (131)			
Parents’ house	55% (558)	50% (260)			
**Degree; % (N)**					
Bachelor’s degree	84.4% (858)	88.6% (455)	χ^2^ = 5.497	1	0.19
Master’s degree	15.5% (158)	11% (57)			
**Year of study; % (N)**					
First year	33.2% (338)^a^	44.6% (229)^b^	χ^2^ = 19.147	3	<0.001
Second year	30% (298)^a^	23.5% (121)^b^			
Third year	21.2% (216)^a^	17.5% (90)^a^			
Fourth year	16% (164)^a^	14% (73)^a^			
Psychotherapy; % (*N*)	11.4% (116)	19.2% (99)	χ^2^ = 17.520	1	<0.001
Medication; % (*N*)	3.5% (36)	7% (36)	χ^2^ = 9.169	1	0.002
**Clinical characteristics**					
Depression (*M* ± SD)	0.337 ± 0.47	0.633 ± 0.48	*t* = 11.473	1,527	<0.001
Risky substance use behavior % (*N*)	31.3% (326)	68.6% (793)	χ^2^ = 33.451	6	<0.001
Identity conflict and acculturation stress (*M* ± SD)	52.366 ± 13.17	58.567 ± 14.049	*t* = 8.496	1,527	<0.001

Items with different superscripts are significantly different at α = 0.017.

As presented in Table 4, there was a significant correlation between NSSI and identity conflict and acculturative stress (*r* = 0.144, *p* < 0.01) as well as risky substance use behavior (*r* = 0.119, *p* < 0.01) and depression (*r* = 0.228, *p* < 0.1). Identity conflict and acculturative stress were significantly correlated to risky substance use behavior (*r* = 0.083, *p* < 0.01) and depression (*r* = −0.360, *p* < 0.01), and risky substance use behaviors were also correlated with depression (*r* = 0.052, *p* < 0.05).

**Table 4:** Pearson correlation matrix of NSSI, demographic and clinical variables.

	**1**	**2**	**3**	**4**	**5**	**6**	**7**	**8**	**9**	**10**	**11**	**12**	**13**	**14**
I.C & Acculturation stress	–	0.083**	0.360**	0.144**	−0.047	−0.013	0.036	−0.002	−0.075**	0.020	−0.057*	−0.005	−0.085**	−0.066**
Risky substance use behavior		–	0.052*	0.119**	−0.012	−0.027	0.046	−0.113**	−0.071**	0.008	0.259**	−0.381**	−0.112**	−0.100
Depression			–	0.282**	−0.056*	−0.053*	−0.050	0.01	−0.064*	−0.033	−0.019	−0.068**	−0.137**	−0.140**
NSSI				–	−0.059*	−0.060*	−0.082**	−0.034	−0.003	0.037	−0.024	−0.091**	−0.107**	−0.077**
Age					–	0.451**	0.338**	0.264**	0.604**	−0.035	−0.012	0.053*	−0.130**	−0.061
Degree						–	−0.064**	0.164*	0.266**	0.019	−0.041	0.026	−0.156**	−0.034
Year							–	0.045	0.159**	−0.019	0.056*	0.044	−0.056*	−0.02
Residence								–	0.244**	0.004	−0.032	0.160**	0.014	−0.014
Marital status									–	−0.003	−0.79**	0.142**	−0.039	0.009
Parents settlement pattern										–	−0.149**	0.058**	−0.011	−0.009
Ethnicity											–	−0.332**	−0.074**	−0.061**
Religion												–	0.091**	0.059*
Psychotherapy													–	0.328**
Medication														–

*p < 0.05, **p < 0.01, ***p < 0.001.

Table 5 presented the results of the logistic regression analysis showing that participants are more likely to engage in NSSI when they also experience depression (*B* = 0.088, *p < 0.*001), identity conflict and acculturative stress (*B* = 0.012, *p* = 0.016) and tend to display risky substance use behavior (*B* = 0.171, *p* = 0.005).

**Table 5:** Logistic regression of correlates of religiosity, age, degree, depression, risky substance use behaviors and identity conflict and acculturation stress.

	*B*	SEB	Wald	*p*	OR (95%CI)
Religiosity	−0.170	0.117	2.084	0.149	0.844 [0.671, 1.063]
Age	−0.016	0.017	0.961	0.327	0.984 [0.953, 1.016]
Degree	−0.186	0.195	0.906	0.341	0.831 [0.567, 1.217]
Depression	0.088	0.011	67.748	>001	1.092 [1.070, 1.115]
Risky substance use behavior	0.171	0.060	8.005	0.005	1.187 [1.054, 1.336]
Identity conflict and acculturation stress	0.012	0.005	5.836	0.016	1.012 [1.002, 1.021]

**Figure 1:**
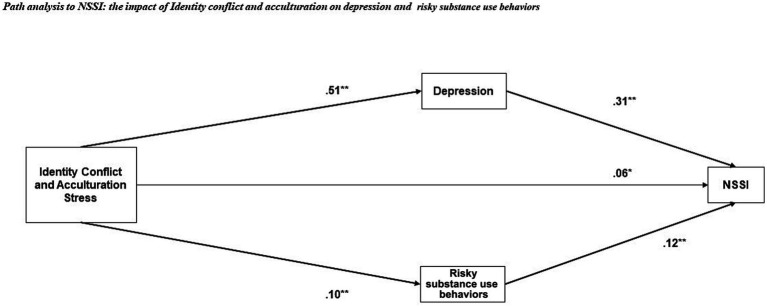
Path model for clinical indicators: NSSI, depression, identity conflict and acculturation stress, and risky substance use behavior. *p < 0.05, **p < 0.01, ***p < 0.001.

Finally, A confirmatory path analysis model to identify pathways to NSSI was conducted. The model has indicated a good fit (CFI = 0.974, TLI = 0.921, NFI = 0.967, RMSEA = 0.05). As hypothesized, there was a significant direct effect of identity conflict and acculturative stress on NSSI (*β* = 0.59*, p* = 0.37, SE = 0.005, 95% [0.010, 0.108]) as well as an indirect effect through its impact on depression (*β* = 0.155, *p* < 0.001, SE = 0.002, 95% [0.011, 0.016]) and risky substance use behavior (*β* = 0.012, *p* < 0.001, SE = 0.001, 95% [0.001, 0.002]) (Figure 1).

## 4. Discussion

The objective of the current study was to estimate the frequency of NSSI and to examine the association of this behavior with identity conflict and acculturative stress, depression, and risky substance use behavior among female Arab students. The results showed high frequencies of engaging in NSSI in this sample compared to other studies (32, 44, 45). In fact, we found that identity conflict and acculturative stress impact the effect of depression and risky substance use behaviors on NSSI.

This finding is consistent with previous findings (46, 47) and may be explained by the association between minority stress and dysregulation, which increases the risk of self-harm. While people engage in NSSI for various reasons, they often use it to regulate intense or unwanted emotions (48). This is supported, for example, by empirical evidence that difficulties in emotion regulation—that is, the processes by which people regulate the characteristics of their emotions—characterize NSSI (49). While the current study did not directly examine emotional dysregulation, previous research has highlighted the association between minority stress, dysregulation, and the risk of self-harm. Additionally, marked differences were found in the emotion regulation of self-injurers compared to non-injurers (50). Moreover, ethnic discrimination events (such as those characterized by minority stress) and fear of stereotyping were found to often cause expressive suppression as an emotionally focused strategy (51, 52). These strategies contribute to problems in self-regulation that could lead to maladaptive coping (53).

Indirectly, minority stress could lead to NSSI due to symptoms of depression, mainly due to racism and discrimination (54, 55). In our study, this is reflected by the fact that most participants with identity conflict and acculturative stress also reported high levels of depression. Our results indicate a relationship between identity conflict, acculturative stress, and depressive symptoms. However, the severity or prevalence of high levels of depression among these participants was not explicitly quantified in our results. The severity of depression may worsen due to the failure to receive professional help (56). In fact, Participants of this study who experience high levels of identity conflict and acculturation stress may exhibit feelings of confusion and uncertainty about their identity, particularly if there is a conflict between one’s original cultural identity and the identity required to navigate the new cultural environment. This can lead to a sense of disconnection from oneself and a lack of belonging, which can contribute to the risk of NSSI as a way of coping with emotional pain.

Acculturation stress may increase the risk of NSSI through its impact on social support. Immigrants and individuals from minority backgrounds may have limited access to social support networks, particularly if they experience discrimination or face language barriers (15, 57). This lack of social support can increase feelings of loneliness and emotional distress, which can contribute to the risk of NSSI.

A study that compared the Arab and Jewish populations in Israel found that of those who reported mental distress, among Arabs, only 14% sought psychotherapy, compared to 36% among Jews (58). The inaccessibility to psychotherapy in Arab society is due to economic, geographic, linguistic, and cultural factors (59). Lack of access to mental health services, rather than cultural barriers such as stigma, was considered to be the main obstacle to help-seeking in this Arab minority population (60). Moreover, Data from the Information Department of the Ministry of Health shows that only 7% of the mental health clinics in Israel operated among the Arab population (61). Specifically, given that previous literature has reported that depression is a valid predictor of NSSI (62, 63) and in view of the findings of the present study, in which the participants were all university students, the current set of services available to meet the needs of Arab citizens students should be reviewed, and academic institutions should consider ways to provide these services.

In times of mental distress and the absence of sources of support, internet use increases (64). While the current study did not directly examine Internet use, it’s worth noting the broader context in which NSSI exists. Previous research indicates that Internet use is increasing remarkably among certain populations suffering from psychosocial problems (65), low self-esteem (66), and difficulties in emotion regulation (67), which, as noted, characterize ethnic minorities. In addition, disadvantaged groups use the Internet to diversify their information sources and social networks (68). Indeed, it was found that the Arab citizens of Israel are more likely than Israeli Jews to search for health information and to communicate online about health-related issues (69). For this particular population (who may be in a state of mental distress and do not receive proper professional help), extensive use of the Internet may expose them to websites that encourage self-harm, as well as to websites that contain technical information on self-harm methods (70). Participants with a history of NSSI were also more likely to engage in risky substance use behavior. Alongside the findings that NSSI and alcohol consumption serve similar emotion regulation functions, alcohol use also increases at the ages when NSSI decreases [e.g., the transition from high school to college; (71)], which suggests that alcohol use could eventually replace NSSI as a means of emotion regulation. As for cannabis use, high THC/CBD levels have been found to cause depression (72), impulsive behaviors (73), and difficulty regulating adverse effects (74). In turn, these conditions are associated with self-injurious behaviors. As noted earlier, these behaviors among ethnic minorities may reflect a stress response to racial/or ethnic stressors (75, 76). Early adulthood is often marked by a greater exploration of one’s social and community identity. Therefore, a minority population may often be subjected to more open manifestations of discrimination and may, in turn, develop a sense of internalized negativity that often leads to coping based on drugs and alcohol (77).

Moreover, although other minorities in Israel were beyond the scope of this study, it is noteworthy that Israel is a unique place to study the mental difficulty of minorities. Due to mass immigration, religious diversity, and a dynamic political, social, and economic environment, the Israeli population is comprised of a growing number of ethnic groups in Israel (78). Although the Arab population constitutes the largest ethnic minority, there are many additional ethnic minority populations, such as Armenians, Cirassians, Assyrians, and some Jewish minority groups. Future work should consider investigating the relationship between identity conflict and NSSI among other ethnic minorities in Israel.

One of the salient aspects of our findings is the data on help-seeking behaviors. Understanding the nuances of how the studied population approaches assistance, especially in mental health, provides a critical perspective into their unique challenges and potential avenues for intervention.

From our results, it is evident that multiple factors influenced the propensity for help-seeking among our participants. Cultural and societal norms, language barriers, perceived stigma, and availability of accessible resources are some potential barriers that might deter individuals from actively seeking help. Understanding these barriers is essential given the high reported levels of identity conflict, acculturative stress, and NSSI. The disparity in the number of participants experiencing distress and those actually seeking help emphasizes the urgent need for more adapted and accessible mental health services for this population. Addressing these barriers could not only enhance the mental well-being of the individuals but also reduce the occurrences of NSSI, which our study highlighted as prevalent in this community.

Furthermore, the focus on help-seeking behaviors may offer knowledge that promotes future interventions that are both culturally sensitive and effective in addressing the unique challenges faced by this population.

### 4.1. Limitations

Several limitations of this study should be noted. Firstly, the measures we used to measure all variables were limited to self-reporting, creating the possibility for a recall and social desirability bias. Secondly, the current study’s generalizability is limited since we did not investigate the differences between the subgroups within the sample. Therefore, further research is needed to investigate differences between the subgroups and perhaps to compare the Arab population in Israel to Arab populations in the MENA countries. Thirdly, this study referred to risky behavior such as alcohol consumption and substance use. Future research should address different types of risky substance use behavior, such as unprotected sexual activity, risky uses of social media, and dangerous driving. Fourthly, our study employed a cross-sectional design, which limits the ability to establish causality. While the associations we observed provide valuable insights, they do not indicate direct causal relationships. A longitudinal study design would be more appropriate to elucidate the temporal dynamics and causality between the variables of interest.

Fifthly, the omission of attention checks in our survey instruments emerges as a notable limitation, compromising our ability to validate the authenticity and attentiveness of participant responses. Although this decision was made to preserve the original structure of established questionnaires, it introduces the potential for inaccurate responses. Future research could explore strategies to maintain instrument validity while also ensuring respondent attentiveness to enhance the robustness of data. Finally, Arab society is a traditional and conservative society characterized by high levels of patriarchal, authoritarian, and religious norms (79). Therefore, certain risky substance use behaviors, such as those we examined in our study, are considered indecent behavior, and constitute social taboos (80, 81). For example, regarding alcohol, the Quran prohibits the consumption of alcohol (82). Therefore, in view of the cultural characteristics of the current study population, there may be a differential bias regarding measures of risky substance use behavior. Presumably, the results would be more accurate and comprehensive if the cultural barriers were controlled.

### 4.2. Implications

Despite these limitations, the strengths of this study—such as the large sample size and that, to the best of our knowledge, it was the first study to examine a relationship between identity conflict and acculturation stress, depression and risky substance use behavior—suggest topics for future research and the development of positive intervention programs focusing on the well-being of the Arab population. Various intervention programs could prevent or reduce the occurrence of depression and NSSI behavior. Such intervention programs include regular monitoring of the students’ mood through daily self-examination in the form of a “mood thermometer” and referral to psychotherapy, if necessary (25), cognitive-behavioral group therapy (83), along with psychoeducation for students and mental health professionals, which includes information about NSSI and how to respond compassionately and effectively to peer disclosures of NSSI (84). In and of itself, awareness might increase sharing and help-seeking, especially given that one of NSSI’s primary functions is the communication of distress.

In light of our findings, several avenues for future research are recommended, including (1) Expanding the population sample: While our investigation is focused on female Arab students, it is essential to consider the experiences of male Arab students. Comprehensive picture of the phenomenon within the broader Arab student population in Israel. (2) As mentioned earlier, Given the cross-sectional nature of our research, a longitudinal approach in subsequent studies could better clarify cause-and-effect relationships, tracking the evolution of identity conflict, acculturation stress, and NSSI over time. (3) Our study established correlations between identity conflict, acculturation stress, depression, risky substance use behavior, and NSSI. A logical next step would be to explore the deeper mechanisms propelling individuals from experiencing these stressors to engaging in NSSI.

## 5. Conclusion

The present study contributes to a growing body of evidence suggesting that ethnic minorities are at increased risk for NSSI. The results of this study highlight the importance of addressing both acculturation stress and depression in order to prevent and treat NSSI, especially among vulnerable populations. Interventions should be culturally appropriate and tailored to the specific needs of individuals from different cultural backgrounds. Early detection and treatment of depression and acculturation stress can help reduce the risk of NSSI and promote positive mental health outcomes.

More specifically, this research may contribute to a broader understanding of the mental distress experienced by the Arab population in Israel, especially among Arab women, since NSSI and depression are still taboo in this population, along with low societal awareness of the phenomenon.

## Data availability statement

The original contributions presented in the study are included in the article/supplementary material, further inquiries can be directed to the corresponding author.

## Ethics statement

The studies involving humans were approved by Academic College of Tel Aviv Yaffo IRB. The studies were conducted in accordance with the local legislation and institutional requirements. The participants provided their written informed consent to participate in this study.

## Author contributions

SM and SH conceived and designed the analysis. SS collected data. SM performed the analysis and original draft preparation. SM, SS, MO, IT, and SH: conceptualization, supervision, and editing. All authors contributed to the article and approved the submitted version.
